# CULTURALLY RESPONSIVE–SUSTAINING EDUCATIONAL STRATEGIES IN UNDERGRADUATE THANATOLOGY COURSES

**DOI:** 10.1093/geroni/igae098.0881

**Published:** 2024-12-31

**Authors:** Autumn Decker, Raven Weaver, Cory Bolkan

**Affiliations:** Pacific University, Hillsboro, Oregon, United States; Washington State University, Pullman, Washington, United States; Washington State University, Pullman, Washington, United States

## Abstract

There is an increasing demand for health and human service (HHS) workers in the United States. With direct and sustained contact with people, HHS workers are regularly exposed to end-of-life (EOL) care but typically have little education and support to handle the associated emotional and mental toll. Thus, this study qualitatively explored how cultural content, cultural humility, and EOL experiences of diverse groups were integrated in undergraduate thanatology courses. In Spring 2023, instructors teaching thanatology courses across the country were asked to participate in a quantitative survey (n=56) and a follow-up 45-minute semi-structured interview (n=27) about the instructor’s course, strategies for addressing diverse populations and content in their course, and effective activities and assignments. Using culturally responsive-sustaining pedagogical principles, interviews with instructors were deductively/inductively coded, resulting in seven themes: 1) Welcoming and Affirming Environment; 2) High Expectations and Rigorous Instruction; 3) Inclusive Curriculum and Assessment; 4) Ongoing Professional Learning; 5) “Doing your Own Work”; 6) Classroom Culture and 7) Desire to Do More. Findings highlight the importance of self-care for instructors, illustrate a need for improved resources and training for death educators, particularly in delivering cultural material; support bringing lived experiences of the instructor into the classroom as a tool for building safety, belongingness, and learning for students; and emphasize the importance of multidisciplinary approach to thanatology. This session will provide tangible suggestions and pedagogical resources for instructors of thanatology courses to encourage using culturally responsive-sustaining principles in the development, delivery, and debrief of in-class activities and assignments.

